# Supplementary material for: The Perceived Impact of Suicide Bereavement on Specific Interpersonal Relationships: A Qualitative Study of Survey Data
Source: Int J Environ Res Public Health. 2019 May 21;16(10):1801. doi: 10.3390/ijerph16101801 (PMC6572476; doi:10.3390/ijerph16101801)
Supplement: Supplementary file 1 [file ijerph-16-01801-s001.pdf]

Table S1. Distribution of themes by relationship type.

		Partner	Close friends	Close family	Wider family	General relationships	Total responses coded under any of the 5 relationship categories
**Themes**							
Social discomfort over the death	n	11	50	43	25	14	143
	%	8%	35%	30%	17%	10%	100%
Social withdrawal	n	63	106	27	0	76	272
	%	23%	39%	10%	0%	28%	100%
Shared bereavement experience creating closeness versus avoidance	n	4	73	36	7	23	143
	%	3%	51%	25%	5%	16%	100%
Attachments influenced by a fear of further losses	n	58	22	74	10	22	186
	%	31%	12%	40%	5%	12%	100%

The numerator represents the number of responses coded under that theme in relation to that specific relationship. The denominator (final column) represents the total number of responses coded under that theme for all relationship categories.
